# Nutritional imbalances among university students and the urgent need for educational and nutritional interventions

**DOI:** 10.3389/fnut.2025.1551130

**Published:** 2025-03-05

**Authors:** Keila Torres, Miguel A. Cáceres-Durán, Constanza Orellana, Martina Osorio, Layla Simón

**Affiliations:** ^1^Escuela de Nutrición y Dietética, Universidad Finis Terrae, Santiago, Chile; ^2^Laboratório de Genética Humana e Médica, Instituto de Ciências Biológicas, Universidade Federal do Pará, Belém, Brazil

**Keywords:** dietary habits, 24-h dietary recall, macronutrient intake, micronutrient intake, adolescents, nutritional deficiency, nutrition recommendations

## Abstract

**Introduction:**

The dietary habits of children and adolescents in Chile have been identified as inadequate, potentially contributing to low levels of essential vitamins and minerals, as well as the development of chronic diseases such as obesity and diabetes. However, the nutritional profile of Chilean university students and the impact of their diets on this profile remain largely unknown. This study aimed to assess the nutritional profile of Finis Terrae University students in the Santiago Metropolitan Region, Chile.

**Methods:**

A retrospective 24-h dietary survey, using the Automated Self-Administered 24-Hour Dietary Assessment Tool (ASA24®), was administered to 124 students between September and October 2024. Data on macronutrient and micronutrient consumption was analyzed using GraphPad Prism.

**Results:**

Among those surveyed, 90% reported consuming at least three meals daily (breakfast, lunch, and dinner). However, 61% consumed fewer calories than recommended, only 7% met the daily intake goals for dairy, 11% met the recommended fruit and vegetable intake, and 16% fiber intake. In contrast, 65% exceeded the upper recommended limits for sodium intake. Significant deficiencies were observed in the intake of calcium, magnesium, potassium, and vitamins A, C, D, and E. Meanwhile, the intake of copper, phosphorus, selenium, folate, niacin, riboflavin, thiamine, vitamin B6, and vitamin B12 met the recommended dietary allowances.

**Discussion:**

Although most of the students consumed three meals daily, the quality of their nutritional intake was suboptimal. These findings highlight the need for educational and nutritional programs to promote healthier eating habits and improve the quality of life of university students, emphasizing regular, balanced meals, developing skills in meal preparation to reduce reliance on ultra-processed foods, and prioritizing hydration with water.

## 1 Introduction

Nutritional status refers to the balance between nutrient intake and the requirements of the body for optimal functioning. It encompasses various factors, including dietary habits, nutrient absorption, and overall health status (1). The increasing prevalence of diet-related noncommunicable diseases (NCDs) such as diabetes, heart disease, stroke, and cancer underscore the urgent need to promote healthy dietary practices. A balanced diet helps prevent malnutrition and reduces the risk of NCDs. However, modern diets, driven by increased consumption of processed foods, urbanization, and changing lifestyles, are now high in energy, fats, sugars, and sodium, with insufficient intake of fiber-rich fruits, vegetables, and whole grains. This shift contributes to poor nutritional status, which can result in deficiencies of essential vitamins and minerals, compromising immune function and increasing susceptibility to illness (2). To counter these trends, global health recommendations emphasize limiting total fat intake to <30% of daily energy, reducing free sugars to <10%, maintaining salt intake below 5 g per day, and prioritizing unsaturated fats over saturated and trans fats. Additionally, diets should incorporate sufficient fruits, vegetables, legumes, and whole grains, which are key to addressing global health risks, particularly among vulnerable populations such as students, who face unique challenges in maintaining balanced nutrition (3, 4).

The university food environment significantly influences the dietary behaviors of students, often promoting unhealthy eating habits due to limited access to healthy options, higher costs for nutritious foods, and the availability of cheaper junk foods. Key factors such as taste, price, and convenience drive the food choices of students, with stress and time constraints exacerbating poor eating habits (5). In Latin American countries, university students who regularly eat breakfast tend to have a more balanced dietary intake compared to those who skip this meal. Consistent breakfast consumption is linked to higher intakes of nutrient-dense foods and healthier choices in later meals, especially dinner (6).

In Chile, dietary habits are unhealthy, particularly among children and adolescents, and are associated with a higher prevalence of obesity and low levels of vitamins and minerals (7–9). For instance, overweight/obesity seems to be the primary condition for vitamin D sequestration. Furthermore, the lack of exposure to sunlight, physical activity or milk consumption are potential causes of vitamin D deficiency in the Chilean population (9). Additionally, the quality of breakfast among Chilean university students has been reported as inadequate due to insufficient fruit consumption and low energy intake (10).

Dietary assessments are essential tools for nutritional diagnosis, intervention planning, and evaluation of consumption patterns. Among these methods, 24-h dietary recalls, which collect detailed information on the type, portion size, and sometimes brand of foods and drinks consumed, accurately represent daily intake, especially when conducted using multiple steps. This information is then coded and processed to calculate energy, nutrient, and other dietary constituent intakes using food composition tables. The selection of an appropriate dietary assessment method depends on the specific purpose of data collection, as each method has distinct strengths and limitations (11, 12).

The Automated Self-Administered 24-Hour (ASA24®) dietary assessment tool, developed by the United States Department of Agriculture, enables respondents to report their food and beverage intake by either browsing predefined food categories or searching through a comprehensive list of specific food and drink items. ASA24® is widely used in research studies globally, offering a valuable tool for dietary assessment (13–15). ASA24® has been validated and has demonstrated accuracy in estimating actual intake, comparable to standard 24-h consumption surveys (16). In this way, the dietary survey is a non-invasive tool that allows data collection to infer a nutritional profile.

The nutritional profile of Chilean university students, particularly those at Finis Terrae University in the Santiago Metropolitan Region, has yet to be thoroughly investigated. Therefore, this study aims to evaluate the dietary habits of these students, identify potential nutritional deficiencies, and propose targeted nutritional recommendations.

## 2 Methods

### 2.1 Sample size calculation

The sample size was calculated as described previously (17–19), using this formula:

$$n=\left[\;Z^{2}\times p\times \left(1-p\right)\times \mathrm{Deff}\;\right]÷\delta^{2}$$

In this sense, *p* is the prevalence of vitamin D deficiency in preadolescents, previously determined in Santiago, Chile (84%) (8); Z is the 95% quantile with a normal distribution (1.96); *δ* is the confidence interval (12%), and Deff = 1.7 is for the simple random sample design effect.

A minimum of 61 was required to detect a medium effect size. Based on this, two independent groups of 62 students were analyzed, resulting in a total of 124 students interviewed. This ensured that each group had a sufficient number of participants to validate the results internally.

### 2.2 Subjects and data collection

This study involved a total of 124 students at Finis Terrae University, Santiago, Chile. Data collection was performed between September (Survey 1, S1, *n* = 62) and October 2024 (Survey 2, S2, *n* = 62). This study was approved by the ethical scientific committee of Finis Terrae University (ID protocol 24-042) and followed the Declaration of Helsinki. All participants provided their written informed consent before their involvement in the study. To ensure a comprehensive analysis of dietary knowledge within the university population, the study included two distinct groups of students. The first group (S1) consisted of first-year students from the Nutrition and Dietetics program (School of Medicine), allowing an assessment of their baseline knowledge before receiving formal training in nutrition. The second group (S2) comprised students from various other faculties, including Medicine, Engineering, Law, Arts, Architecture and Design, Economics, Education and Psychology, Humanities, and Communication, providing a broader perspective on general dietary knowledge among university students. The inclusion criteria were being students at Finis Terrae University, staying at the university campus or classrooms, accepting voluntarily to participate in the study through informed consent, and having time and availability to respond to the recall. The exclusion criteria were being unavailable to make the recall. All the eligible participants satisfied the above criteria and were included in this study.

### 2.3 Nutritional intake

The automated self-administered 24-h (ASA24®) dietary assessment tool version 2024 available in Spanish (20) was used to assess dietary intake. The survey captured the food consumption of participants over 24 h (12:00 am to 11:59 pm) through recall on a web-based platform, with assistance provided by a researcher trained in the tool. The dietary assessment tool covered all meals (breakfast, lunch, and dinner), snacks, and drinks consumed between meals, including supplements, with additional details for each. On average, the survey took 30 min to complete. Two researchers independently reviewed each completed survey. Finally, detailed daily macronutrient (carbohydrates, proteins, fats) and micronutrient (vitamins, minerals) intake information was obtained based on the foods and beverages consumed, including portion sizes, and reported food items. ASA24® nutrition guidelines are based on calorie and food group recommendations from the Dietary Guidelines for Americans and nutrient requirements established by the Institute of Medicine, National Academy of Sciences (20). We applied the ASA24® dietary recall to two independent groups [survey 1 (*n* = 62) and 2 (*n* = 62)] to internally validate and characterize a usual dietary intake.

### 2.4 Statistical analysis

All statistical analyses were conducted using the GraphPad Prism program, applying the ANOVA test, linear regression, and correlation analyses. A *p* < 0.001 was considered a statistically significant difference.

## 3 Results

### 3.1 Meal consumption patterns

**Figure 1:**
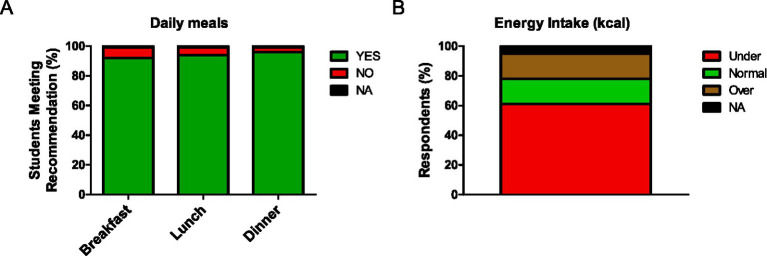
**(A)** Consumption of the three main daily meals (breakfast, lunch, and dinner) among students surveyed at Finis Terrae University, Chile. **(B)** Percentage of respondents reporting energy intake at recommended levels (green), under (red), or over recommended levels (brown). Not applicable (NA) represents respondents who did not report their intake. *n* = 124.

Our results indicate that the majority of students maintained regular meal consumption, with 90% of respondents reporting the intake of at least three daily meals: breakfast, lunch, and dinner (Figure 1A). However, 61% of the respondents consumed less energy than recommended (Figure 1B), suggesting a poor quality or quantity of their daily meals. Similar results were observed in surveys 1 and 2 (Supplementary Figure 1), suggesting a validation of the results and a good characterizing of the usual dietary intake.

**Figure 2:**
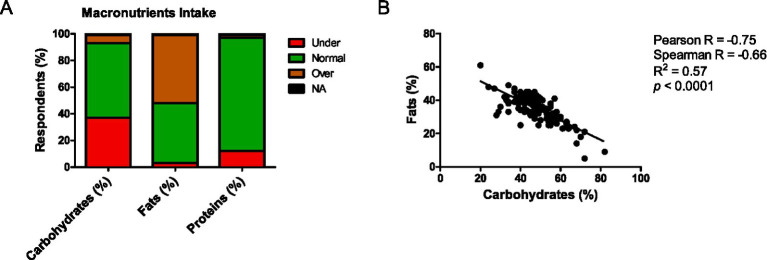
**(A)** Percentage of respondents reporting macronutrient intake at recommended levels (green), under (red), or over recommended levels (brown). Not applicable (NA) represents respondents who did not report their intake. **(B)** Correlation between percentage of fat *vs* carbohydrate intake. *n* = 124.

Although only 10% of students failed to eat some meals, significant discrepancies in macronutrient intake were observed. As shown in Figure 2A, 37% of students consumed fewer carbohydrates than the recommended. Additionally, 51% of students consumed more fat than recommended. In this sense, some respondents have a notable preference for fat consumption over carbohydrates in both surveys (Figure 2A; Supplementary Figure 2). These data highlight a strong inverse association between over-consumed fat and under-consumed carbohydrates (Figure 2B). Altogether, these results indicate that a considerable proportion of students either under-consume or over-consume critical nutrients, highlighting potential nutritional imbalances.

**Figure 3:**
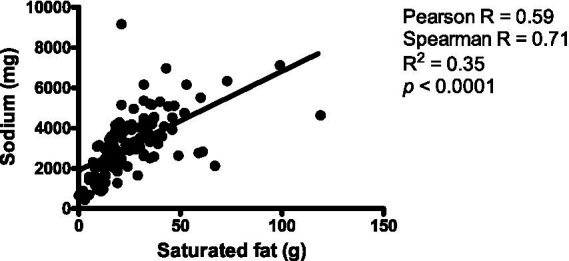
Correlation between saturated fat and sodium intake (*n* = 124).

Table 1 summarizes the intake of nutrients to limit, revealing moderately high consumption of added sugars and saturated fats. Particularly concerning is sodium intake, with 65% of respondents exceeding the maximum recommended limit, while only 35% met the guidelines. These findings were similar between surveys 1 and 2 (Supplementary Table 1). Additionally, there was a moderate positive association between saturated fat and sodium consumption (Figure 3), indicating that as saturated fat consumption increases, sodium intake tends to increase as well.

**Table 1:** Intake of added sugars, saturated fat, and sodium compared to upper recommended limits.

Nutrient	Intake	Upper Limit	Percentage of students meeting recommendation	*p-*value
Added sugars (g/d)	57 ± 48	57 ± 7	65%	ns
Saturated fat (g/d)	26 ± 18	25 ± 3	69%	ns
Sodium (mg/d)	3,170 ± 1,468 ^a^	2,300 ^b^	35%	*p* < 0.001

Mean ± Standard Deviation; ns, not significant; different letters indicate statistically significant differences (*p* < 0.001); *n* = 124.

### 3.2 Food group, vitamin and mineral intake

Table 2 highlights significant deficiencies in the intake of essential food groups, such as fruits, dairy, and vegetables. Only 7% of students met the recommended intake for dairy, 11% met the recommended intake of fruits and vegetables, and 16% achieved the recommended intake for total fiber. The results of both surveys had a similar tendency (Supplementary Table 2).

**Table 2:** Food group intake, recommended values, and the percentage of the students meeting the recommendations.

Nutrient	Intake	Recommendation	Percentage of students meeting recommendation	*p-*value
Grains (ounces)	7 ± 4	7 ± 2	50%	ns
Fruits (cups)	1 ± 1 ^a^	2 ± 0 ^b^	11%	*p* < 0.001
Dairy (cups)	1 ± 1 ^a^	3 ± 0 ^b^	7%	*p* < 0.001
Vegetables (cups)	1 ± 1 ^a^	3 ± 0 ^b^	11%	*p* < 0.001
Total Fiber (g)	14 ± 8 ^a^	28 ± 6 ^b^	16%	*p* < 0.001
Proteins (g)	89 ± 84	48 ± 4	84%	ns

Mean ± Standard Deviation; ns, not significant; different letters indicate statistically significant differences (*p* < 0.001); *n* = 124.

Dairy products were the main protein sources commonly consumed among the students (consumed by 70% of students), followed by red meat, chicken, pig, and legumes (35%). Fish and eggs were less consumed (20%).

Our findings reveal that although students achieved a high percentage of the recommended intake for folate, niacin, riboflavin, thiamine, vitamin B6, vitamin B12, and K, significant deficiencies were observed in other vitamins (Table 3). Specifically, vitamin D intake was markedly insufficient, with only 3% of students meeting the recommended daily allowance. Furthermore, the intake of vitamins E, A, and C was inadequate, with only 14, 19, and 29% of students reaching the recommended levels, respectively. In the case of niacin, riboflavin, thiamine, vitamin B6 and vitamin B12 intake was significantly higher than the recommendation.

**Table 3:** Vitamin intake, recommended values, and percentage of the students meeting the recommendation.

Vitamin	Intake	Recommendation	Percentage of students meeting recommendation	*p*-value
Folate (mcg)	504 ± 291	400 ± 0	69%	ns
Niacin (Vitamin B3) (mg)	27 ± 15 ^a^	15 ± 1 ^b^	87%	*p* < 0.001
Riboflavin (Vitamin B2) (mg)	2 ± 1 ^a^	1 ± 0 ^b^	86%	*p* < 0.001
Thiamine (Vitamin B1) (mg)	2 ± 1 ^a^	1 ± 0 ^b^	81%	*p* < 0.001
Vitamin A (mcg)	435 ± 328 ^a^	748 ± 86 ^b^	19%	*p* < 0.001
Vitamin B6 (mg)	2 ± 2 ^a^	1 ± 0 ^b^	69%	*p* < 0.001
Vitamin B12 (mcg)	5 ± 3 ^a^	2 ± 0 ^b^	66%	*p* < 0.001
Vitamin C (mg)	54 ± 61 ^a^	77 ± 9 ^b^	29%	*p* < 0.001
Vitamin D (IU)	167 ± 177 ^a^	600 ± 0 ^b^	3%	*p* < 0.001
Vitamin E (mg)	7 ± 5^a^	15 ± 0 ^b^	14%	*p* < 0.001
Vitamin K (mcg)	106 ± 163	94 ± 14	42%	ns

Mean ± Standard Deviation; ns, not significant; different letters indicate statistically significant differences (*p* < 0.001); *n* = 124.

The data presents a mixed consumption pattern for minerals (Table 4). Copper, iron, and zinc intakes are close to the recommended levels, with 65, 46, and 62% of students meeting the recommendation, respectively. However, calcium, magnesium, and potassium consumption were significatively deficient, with only 28, 26, and 36% of respondents meeting the recommended intake, respectively. In the case of phosphorus and selenium intake was significantly higher than the recommendation. The results of both surveys had a similar tendency in terms of vitamin and mineral intake (Supplementary Tables 3, 4).

**Table 4:** Mineral intake, recommended values, and percentage of the recommendation met.

Mineral	Intake	Recommendation	Percentage of students meeting recommendation	*p*-value
Calcium (mg)	757 ± 536^a^	1029 ± 160^b^	28%	*p* < 0.001
Copper (mg)	1 ± 0	1 ± 0	65%	ns
Iron (mg)	14 ± 7	15 ± 4	46%	ns
Magnesium (mg)	246 ± 131^a^	339 ± 39^b^	26%	*p* < 0.001
Phosphorus (mg)	1193 ± 606^a^	784 ± 199^b^	80%	*p* < 0.001
Potassium (mg)	2152 ± 1020^a^	2641 ± 449^b^	36%	*p* < 0.001
Selenium (mcg)	128 ± 69^a^	55 ± 0^b^	90%	*p* < 0.001
Zinc (mg)	11 ± 8	9 ± 1	62%	ns

Mean ± Standard Deviation; ns, not significant; different letters indicate statistically significant differences (*p* < 0.001); *n* = 124.

## 4 Discussion

This study aimed to assess the dietary habits and nutrient intake of students at Finis Terrae University, Chile. Our findings revealed that 90% of students consumed three meals daily, with <10% skipping breakfast. These results are significantly higher than those reported by Díaz-Torrente et al. (10), where only 53% of Chilean university students consumed breakfast regularly. Additionally, all students reported dinner consuming, which may be a positive result. A large retrospective cohort study in Japan found that skipping dinner was significantly associated with a ≥ 10% increase in body weight and a higher risk of overweight/obesity in both male and female students (21). However, our data on energy, macronutrient, and micronutrient intakes suggest that the quality and quantity of these meals are suboptimal. Specifically, we find that the intake of fruits, vegetables, dairy, and total fiber is below the recommended levels. This finding aligns with observations in the Chilean general population, where 90% consume less fiber than recommended (minimum intake of 25 g/day) (22) and the quality and quantity of meals are inadequate (10).

The current investigation reveals a significant dietary imbalance among university students, characterized by excessive sodium, but insufficient vitamins and minerals intake. In the general Chilean population, sodium intake is substantially higher than recommended, with an average consumption of ≥9 g of salt per day (23). Petermann-Rocha et al. (23) also found that elevated sodium excretion levels are more common among males, older adults, individuals with overweight or obesity, and those with hypertension, metabolic syndrome, or diabetes. Notably, the risk of hypertension rises markedly when sodium excretion reaches 3.2 g/day. This finding aligns with Wu et al. (24), who reported high sodium consumption among Chinese college students, with an average intake of approximately 3 grams of salt per day. Similarly, our results indicate a positive correlation between saturated fat and sodium consumption, likely associated with the frequent intake of fast food and ultra-processed products.

Additionally, we observed deficiencies in vitamins A, C, E, and D, calcium, magnesium, and potassium, and nutrient gaps that could pose long-term health risks, particularly affecting bone health and energy metabolism (25). In Chile, vitamin D (25-hydroxyvitamin D) deficiency is more prevalent among men and older adults, with pronounced seasonal variation during the winter and spring (26). A decrease in serum vitamin D levels has been correlated with higher rates of overweight and obesity among Chilean children (9). Our results are aligned with a study conducted on 585 younger health sciences students in Spain, where dietary intake, assessed via a 72-h recall, showed similar nutritional imbalances, and deficient levels of essential nutrients such as iodine and vitamins D and E. Comparable patterns have also been observed in the general population of Spain (27).

Understanding the nutritional behaviors of university students during this formative period is essential for identifying gaps in both theoretical knowledge and practical application of healthy eating habits. As reported by Whatnall et al. (28) there is a positive association between healthier dietary intake and academic achievement. Notably, external factors significantly influence university students. University students, especially those living independently, often face increased financial and time pressures, leading to fewer healthy eating habits. Limited access to nutritious options, the prevalence of convenient but unhealthy foods, and inadequate cooking facilities make it challenging for students to maintain a balanced diet, pushing them toward easier, less healthy choices (29). The transition from high school to university is frequently associated with marked lifestyle changes, including reductions in physical activity, weight gain, and significant dietary shifts. Notably, declines in the consumption of fruits, vegetables, and dairy products have been widely reported during this stage (30).

Fonseca et al. (31) identified distinct dietary patterns among Brazilian university students, largely shaped by the campus food environment, where items like jam, chocolate drinks, and processed juice contribute substantially to their eating habits. Similarly, poor diet quality was identified among both domestic and international students at an Australian university. Food insecurity was linked to reduced diet quality in domestic students; however, the effect on international students remained inconclusive due to the limited sample size (32). In Lebanon, students demonstrated moderate but inconsistent adherence to the Mediterranean Diet, with higher adherence observed among older students and nonsmokers (33).

Furthermore, a study of 535 nursing students at the University of Castilla-La Mancha identified significant links between food addiction and lifestyle factors such as a sedentary lifestyle, anxiety or depression, and poor sleep quality (34). Low fish consumption appears to correlate with a higher incidence of depression among Spanish university students aged 18 and older, further underscoring the role of dietary choices in health outcomes (35).

In China, an assessment of 1,900 undergraduate students showed a higher prevalence of obesity among males compared to females; however, female students demonstrated higher rates of overeating and insufficient physical activity (36). Tao et al. (37) reported that university students in Macao, China, exhibit a dietary pattern characterized by higher consumption of animal foods and lower intake of soy, nuts, and dairy. Notably, the intake of essential nutrients among these students, including vitamin A, thiamine, calcium, and iodine, was significantly below the recommended nutrient intake for China.

Nonetheless, certain limitations in our study warrant consideration. Although we reached the proposed sample size for 24-h dietary recall among two independent groups of students at Finis Terrae University in Santiago, Chile, this sampling approach may limit the generalizability of our findings to a broader population. Additionally, while the ASA24® tool effectively estimates mean total energy and protein intakes, these values tend to be slightly lower than those obtained from recovery biomarkers (14, 15). Furthermore, a drawback of the tool is its reliance on repetitive prompts and detailed question formats, which may impose a significant cognitive load on users, potentially impacting the accuracy and ease of dietary recall (38). However, 85% of respondents in this study found it easy and technically feasible to assess their nutritional profile using the ASA24® tool. Future research should include diverse student populations and employ comprehensive dietary assessment tools to enhance our understanding of dietary challenges and opportunities for health promotion among university students.

### 4.1 Nutritional policies in Chile and recommendations for student health and wellbeing

Various national initiatives in Chile aim to enhance nutrition and improve the overall quality of life for citizens. The mandated fortification of milk with vitamin D is being progressively implemented to combat widespread deficiencies in this essential nutrient. This initiative seeks to reduce the health risks associated with low vitamin D levels, which can lead to conditions such as osteoporosis and weakened immune function (39). Additionally, the Food Scholarship for Higher Education [known as BAES: Beca (Scholarship) de Alimentación (Food) para la Educación Superior (Higher Education)] program represents a significant effort to promote healthy eating habits among university students by providing financial support for nutritious food options (40). Chile has also made strides in implementing front-of-package (FoP) nutritional warning labels, as mandated by the 2016 Food Labeling and Advertising Law. This regulation requires warning labels on products with excessive amounts of nutrients of concern, such as sugars, saturated fats, and sodium. Experimental research demonstrated that such warnings significantly decrease consumer preference and purchase likelihood for products high in sugar and other harmful nutrients, proving the impact of FoP labels on promoting healthier choices (41). Another outcome of the law has been an increased use of non-nutritive sweeteners (NNS) in reformulated products aiming to avoid warning labels by lowering sugar content. Research indicates that the prevalence of NNS products rose from 37.9 to 43.6% after the implementation of the law, with sucralose, acesulfame-K, aspartame and stevia being the most commonly used, followed by saccharin and cyclamate (42).

Despite these commendable efforts, there is still a pressing need for further initiatives to improve the dietary habits of children and adolescents in Chile, as these groups are particularly vulnerable to the impacts of poor nutrition. For instance, the mandated fortification of dairy products with vitamin D needs to be aligned with education on the benefits of consuming dairy products which may improve milk and vitamin D intake. In addition, BAES program needs to be associated with better knowledge of food selection. Addressing this challenge may benefit from following the 10 guiding messages for food culture in Chile by the Chilean Ministry of Health (43), which emphasize the importance of choosing fresh, locally sourced foods from markets and fairs, and enriching meals with various colorful fruits and vegetables. The messages advocate for the frequent consumption of legumes in dishes and salads and drinking water throughout the day instead of sugary beverages. Dairy products are encouraged at all life stages, and it is recommended to increase the intake of fish, seafood, and seaweed from authorized sources. Additionally, the importance of avoiding ultra-processed foods with high-fat or high-sugar labels is stressed. Engaging in shared cooking tasks fosters enjoyment of both traditional and new recipes. Mealtime should be a mindful experience, ideally enjoyed with others and free from screens. Finally, there is an emphasis on environmental responsibility: protecting the planet by conserving water, minimizing food waste, separating waste, and recycling. These principles promote nutritious, safe, and sustainable foods, support sustainable food systems, prioritize fresh and minimally processed options, acknowledge the diversity of local food production, appreciate the value of home cooking, and respect cultural food practices (43).

## 5 Conclusion

In conclusion, while students at Finis Terrae University in Chile generally meet recommended intakes for proteins and certain vitamins and minerals, their overall dietary patterns reveal significant gaps. Specifically, there is an insufficient consumption of key food groups, such as fruits, vegetables, fibers, and dairy products, alongside notable deficiencies in essential nutrients, including vitamins A, C, D, and E, calcium, magnesium, and potassium. This imbalance suggests that although one group in this study consisted of first-year students from the Nutrition and Dietetics program, who have a rudimentary understanding of nutrition principles, they may still face challenges in applying these concepts to their own dietary choices, potentially due to academic demands, lifestyle adjustments, and limited practical skills in meal planning.

Addressing these gaps through enhanced nutritional education, including practical workshops on meal preparation, budgeting, and time management, could greatly benefit all university students. Additionally, integrating counseling and peer support on healthy eating habits into the curriculum might help them make more informed food choices that align with their nutritional knowledge. Overall, these improvements would foster a more balanced nutrient intake and serve as a foundation for lifelong healthy eating habits, ensuring that nutrition students exemplify the principles they aim to promote in their future professional careers. This underscores the need for enhanced nutritional education and potential dietary adjustments to ensure a more balanced intake of essential nutrients.

## Data Availability

The raw data supporting the conclusions of this article will be made available by the authors, without undue reservation.

## References

[1] Gibson RS. Principles of nutritional assessment. 2nd ed. New York, NY: Oxford University Press (2005).

[2] Morales F Montserrat-de la Paz S Leon MJ Rivero-Pino F. Effects of malnutrition on the immune system and infection and the role of nutritional strategies regarding improvements in Children’s health status: a literature review. Nutrients. (2023) 16:1. doi: 10.3390/nu16010001, PMID: 38201831 PMC10780435

[3] Pan American Health Organization. (2024). Nutrition. Available online at: https://www.paho.org/en/topics/nutrition (accessed October 8, 2024).

[4] Al Jawaldeh A. Healthy diet/World Health Organization. Geneva: WHO (2019).

[5] Li X Braakhuis A Li Z Roy R. How does the university food environment impact student dietary behaviors? A systematic review. Front Nutr. (2022) 9:840818. doi: 10.3389/fnut.2022.840818, PMID: 35571951 PMC9090611

[6] Saavedra Clarke S Parra-Soto S Murillo G Carpio-Arias V Landaeta-Díaz L Nava-González EJ. Self-reported nutritional status and breakfast characterization in Latin American University students. J Am Nutr Assoc. (2023) 43:252–60. doi: 10.1080/27697061.2023.2263526, PMID: 37800672

[7] Bustos-Arriagada E Fuentealba-Urra S Etchegaray-Armijo K Quintana-Aguirre N Castillo-Valenzuela O. Feeding behaviour and lifestyle of children and adolescents one year after lockdown by the COVID-19 pandemic in Chile. Nutrients. (2021) 13:4138. doi: 10.3390/nu13114138, PMID: 34836396 PMC8625155

[8] Castillo-Valenzuela O Duarte L Arredondo M Iñiguez G Villarroel L Pérez-Bravo F. Childhood obesity and plasma micronutrient deficit of Chilean children between 4 and 14 years old. Nutrients. (2023) 15:1707. doi: 10.3390/nu15071707, PMID: 37049547 PMC10096594

[9] Pérez-Bravo F Duarte L Arredondo-Olguín M Iñiguez G Castillo-Valenzuela O. Vitamin D status and obesity in children from Chile. Eur J Clin Nutr. (2022) 76:899–901. doi: 10.1038/s41430-021-01043-9, PMID: 34773092 PMC9187513

[10] Díaz-Torrente X Quintiliano-Scarpelli D. Dietary patterns of breakfast consumption among Chilean university students. Nutrients. (2020) 12:552. doi: 10.3390/nu12020552, PMID: 32093261 PMC7071493

[11] Bailey RL. Overview of dietary assessment methods for measuring intakes of foods, beverages, and dietary supplements in research studies. Curr Opin Biotechnol. (2021) 70:91–6. doi: 10.1016/j.copbio.2021.02.007, PMID: 33714006 PMC8338737

[12] Dao MC Subar AF Warthon-Medina M Cade JE Burrows T Golley RK. Dietary assessment toolkits: an overview. Public Health Nutr. (2019) 22:404–18. doi: 10.1017/S1368980018002951, PMID: 30428939 PMC6368251

[13] Subar AF Kirkpatrick SI Mittl B Zimmerman TP Thompson FE Bingley C. The automated self-administered 24-hour dietary recall (ASA24): a resource for researchers, clinicians, and educators from the National Cancer Institute. J Acad Nutr Diet. (2012) 112:1134–7. doi: 10.1016/j.jand.2012.04.016, PMID: 22704899 PMC3721511

[14] Moshfegh AJ Rhodes DG Baer DJ Murayi T Clemens JC Rumpler WV. The US Department of Agriculture Automated Multiple-Pass Method reduces bias in the collection of energy intakes. Am J Clin Nutr. (2008) 88:324–32. doi: 10.1093/ajcn/88.2.324, PMID: 18689367

[15] Kipnis V. Structure of dietary measurement error: results of the OPEN biomarker study. Am J Epidemiol. (2003) 158:14–21. doi: 10.1093/aje/kwg091, PMID: 12835281

[16] Park Y Dodd KW Kipnis V Thompson FE Potischman N Schoeller DA. Comparison of self-reported dietary intakes from the automated self-administered 24-h recall, 4-d food records, and food-frequency questionnaires against recovery biomarkers. Am J Clin Nutr. (2018) 107:80–93. doi: 10.1093/ajcn/nqx002, PMID: 29381789 PMC5972568

[17] Duffau TG. Tamaño muestral en estudios biomédicos. Rev Chil Pediatr. (1999) 70:9. doi: 10.4067/S0370-41061999000400009

[18] Romero-Martínez M Shamah-Levy T Cuevas-Nasu L Méndez Gómez-Humarán I Gaona-Pineda EB Gómez-Acosta LM. Diseño metodológico de la Encuesta Nacional de Salud y Nutrición de Medio Camino 2016. Salud Publica Mex. (2017) 59:299. doi: 10.21149/8593, PMID: 28902317

[19] Cochran WG. Técnicas de muestreo. CECSA. (1985).

[20] NIH. Automated self-administered 24-hour (ASA24®) dietary assessment tool. Available online at: https://epi.grants.cancer.gov/asa24/ (Accessed November 2, 2024).

[21] Yamamoto R Tomi R Shinzawa M Yoshimura R Ozaki S Nakanishi K. Associations of skipping breakfast, lunch, and dinner with weight gain and overweight/obesity in university students: a retrospective cohort study. Nutrients. (2021) 13:271. doi: 10.3390/nu13010271, PMID: 33477859 PMC7832851

[22] Guzmán-Pincheira C Espinoza J Durán-Agüero S Obregón AM Fuentealba F. Dietary fibre intake in Chile: 13 years after the last National Report. Nutrients. (2023) 15:3671. doi: 10.3390/nu15173671, PMID: 37686702 PMC10490374

[23] Petermann-Rocha F Sillars A Brown R Sweeney L Troncoso C García-Hermoso A. Sociodemographic patterns of urine sodium excretion and its association with hypertension in Chile: a cross-sectional analysis. Public Health Nutr. (2019) 22:2012–21. doi: 10.1017/S1368980018003889, PMID: 30761970 PMC10260645

[24] Wu M Xi Y Huo J Xiang C Yong C Liang J. Association between eating habits and sodium intake among Chinese university students. Nutrients. (2023) 15:1570. doi: 10.3390/nu15071570, PMID: 37049412 PMC10097125

[25] Sizar O Khare S Goyal A Givler A. Vitamin D Deficiency. Treasure Island, FL: StatPearls Publishing (2023). 30335299

[26] Vallejo MS Blümel JE Arteaga E Aedo S Tapia V Araos A. Gender differences in the prevalence of vitamin D deficiency in a southern Latin American country: a pilot study. Climacteric. (2020) 23:410–6. doi: 10.1080/13697137.2020.1752171, PMID: 32367772

[27] Correa-Rodríguez M Pocovi G Schmidt-RioValle J González-Jiménez E Rueda-Medina B. Assessment of dietary intake in Spanish university students of health sciences. Endocrinol Diabetes Nutr. (2018) 65:265–73. doi: 10.1016/j.endinu.2018.01.005, PMID: 29599102

[28] Whatnall MC Patterson AJ Burrows TL Hutchesson MJ. Higher diet quality in university students is associated with higher academic achievement: a cross-sectional study. J Hum Nutr Diet. (2019) 32:321–8. doi: 10.1111/jhn.12632, PMID: 30810252

[29] Maillet MA Grouzet FME. Understanding changes in eating behavior during the transition to university from a self-determination theory perspective: a systematic review. J Am Coll Heal. (2023) 71:422–39. doi: 10.1080/07448481.2021.1891922, PMID: 34292133

[30] Winpenny EM Smith M Penney T Foubister C Guagliano JM Love R. Changes in physical activity, diet, and body weight across the education and employment transitions of early adulthood: a systematic review and meta-analysis. Obes Rev. (2020) 21:12962. doi: 10.1111/obr.12962, PMID: 31955496 PMC7079102

[31] Fonseca LB Pereira LP Rodrigues PRM Andrade Ac De S Muraro AP Gorgulho BM. Food consumption on campus is associated with meal eating patterns among college students. Br J Nutr. (2021) 126:53–65. doi: 10.1017/S0007114520003761, PMID: 32967740

[32] Shi Y Grech A Allman-Farinelli M. Diet quality among students attending an Australian university is compromised by food insecurity and less frequent intake of home cooked meals. A cross-sectional survey using the validated healthy eating index for Australian adults (HEIFA-2013). Nutrients. (2022) 14:4522. doi: 10.3390/nu14214522, PMID: 36364787 PMC9655026

[33] Karam J Bibiloni MDM Serhan M Tur JA. Adherence to Mediterranean diet among Lebanese University students. Nutrients. (2021) 13:264. doi: 10.3390/nu13041264, PMID: 33921397 PMC8069129

[34] Romero-Blanco C Hernández-Martínez A Parra-Fernández ML Onieva-Zafra MD Del C P-LM Rodríguez-Almagro J. Food addiction and lifestyle habits among university students. Nutrients. (2021) 13:1352. doi: 10.3390/nu13041352, PMID: 33919610 PMC8073513

[35] Morales-Suárez-Varela M Amezcua-Prieto C Llopis-Gonzalez A Ayan Perez C Mateos-Campos R Hernández-Segura N. Prevalence of depression and fish consumption among first year Spanish university students: UniHcos project. Nutrients. (2023) 15:2757. doi: 10.3390/nu15122757, PMID: 37375661 PMC10300875

[36] Hao M Yang J Xu S Yan W Yu H Wang Q. The relationship between body dissatisfaction, lifestyle, and nutritional status among university students in southern China. BMC Psychiatry. (2023) 23:705. doi: 10.1186/s12888-023-05215-8, PMID: 37777718 PMC10543264

[37] Tao X Shao Y Xu D Huang Y Yu X Zhong T. Dietary patterns and nutrient intake in university students of Macao: a cross-sectional study. Nutrients. (2022) 14:3642. doi: 10.3390/nu14173642, PMID: 36079899 PMC9460302

[38] Mackenzie KM Kerr DA Whitton C Talati Z McCaffrey TA Mullan BA. Predicting perceived problems in self-administered 24-hour dietary recalls: a quantitative think-aloud study comparing automated self-assisted 24-hour dietary assessment tool (ASA24®) and INTAKE24© in university students. Nutrients. (2022) 14:4281. doi: 10.3390/nu14204281, PMID: 36296964 PMC9607278

[39] Ministerio De Salud. Subsecretaría de salud pública. Modifica decreto supremo n° 977, de 1996, del ministerio de salud, reglamento sanitario de los alimentos. Available online at: https://www.bcn.cl/leychile/navegar?idNorma=1204340 (Accessed November 9, 2024).

[40] Ministerio de Educación. Beca de Alimentación (BAES). Available online at: https://portal.beneficiosestudiantiles.cl/becas-y-creditos/beca-de-alimentacion-baes (Accessed November 9, 2024).

[41] Reyes M Garmendia ML Olivares S Aqueveque C Zacarías I Corvalán C. Development of the Chilean front-of-package food warning label. BMC Public Health. (2019) 19:906. doi: 10.1186/s12889-019-7118-1, PMID: 31286910 PMC6615240

[42] Zancheta Ricardo C Corvalán C Smith Taillie L Quitral V Reyes M. Changes in the use of non-nutritive sweeteners in the Chilean food and beverage supply after the implementation of the food labeling and advertising law. Front Nutr. (2021) 8:773450. doi: 10.3389/fnut.2021.773450, PMID: 34859036 PMC8630583

[43] Bustos-Zapata N Varela Barrientos M. Guías Alimentarias para Chile. 2nd ed. Santiago: Ministerio de Salud Subsecretaría de Salud Pública División de Políticas Públicas Saludables y Promoción Departamento de Nutrición y Alimentos (2022).

