# Supplementary figures and images for: Nutritional imbalances among university students and the urgent need for educational and nutritional interventions
Source: Front Nutr. 2025 Mar 5;12:1551130. doi: 10.3389/fnut.2025.1551130 (PMC11919654; doi:10.3389/fnut.2025.1551130)

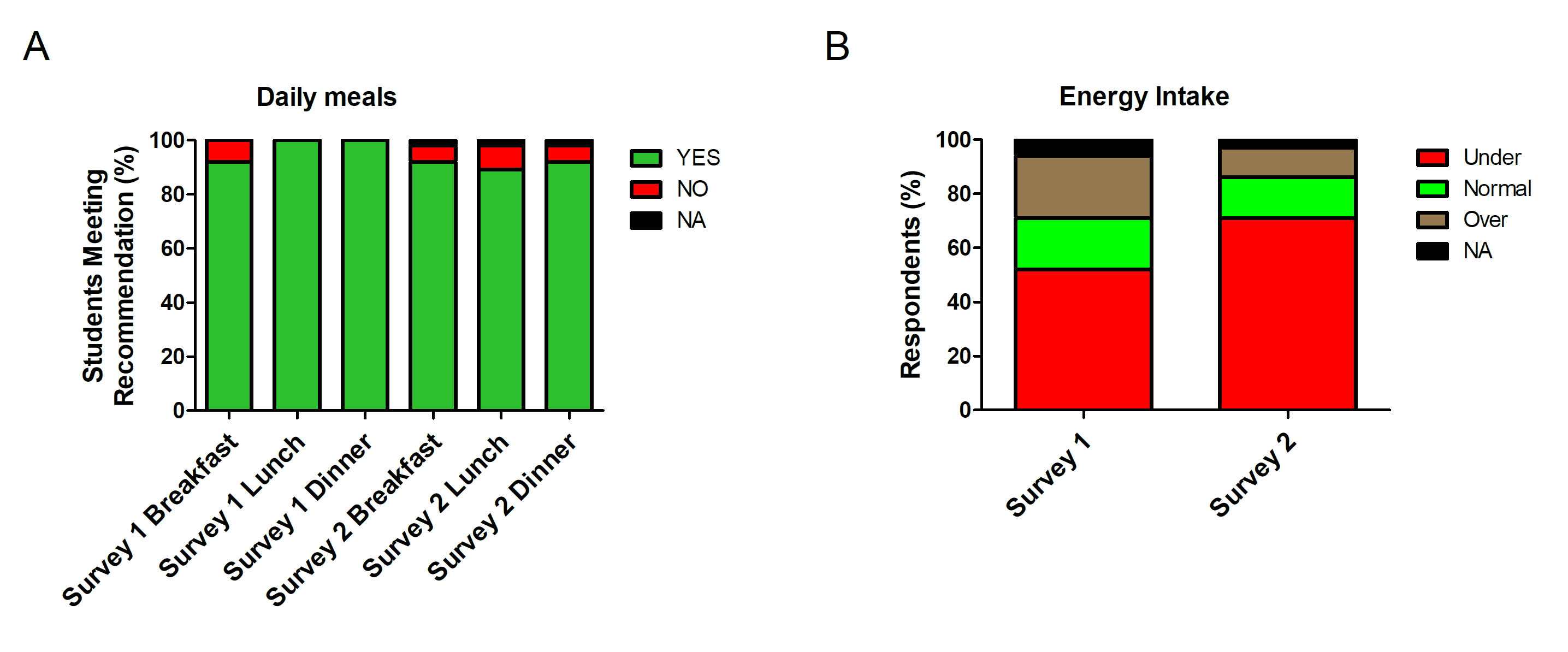

Supplement: Supplementary file 1 [file Image_1.tif]

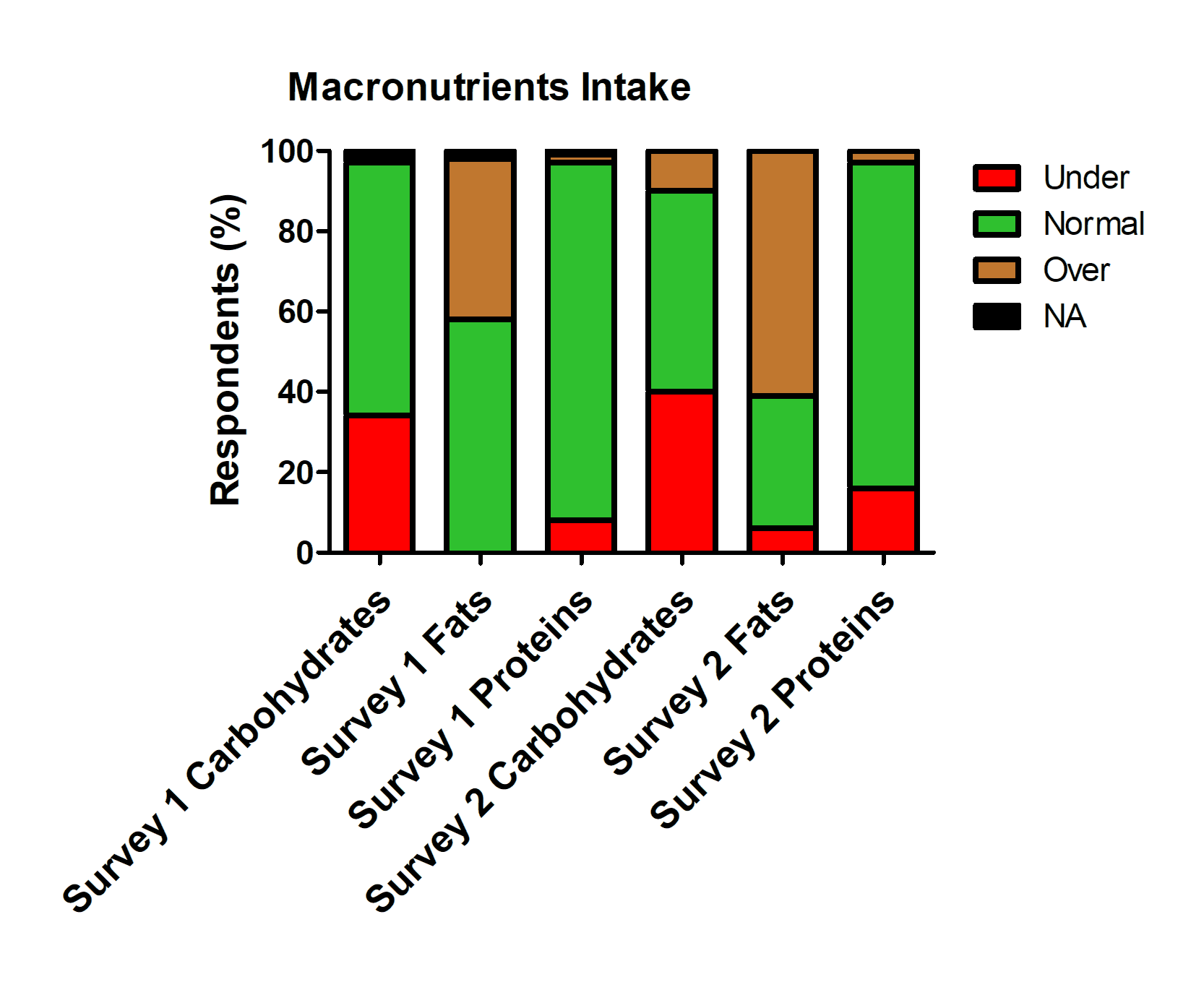

Supplement: Supplementary file 2 [file Image_2.tif]
