# Supplementary material for: Nutritional imbalances among university students and the urgent need for educational and nutritional interventions
Source: Front Nutr. 2025 Mar 5;12:1551130. doi: 10.3389/fnut.2025.1551130 (PMC11919654; doi:10.3389/fnut.2025.1551130)
Supplement: Supplementary file 3 [file Table_1.docx]

Supplementary Material

# Supplementary Tables

**Supplementary Table 1**

Intake of added sugars, saturated fat, and sodium compared to upper recommended limits.

|  | **Survey 1 (n = 62)** | | | **Survey 2 (n = 62)** | | |  |
| --- | --- | --- | --- | --- | --- | --- | --- |
| **Nutrient** | **Intake** | **Upper Limit** | **Percentage of Students Meeting Recommendation** | **Intake** | **Upper Limit** | **Percentage of Students Meeting Recommendation** | ***p-*value** |
| Added Sugars (g/d) | 62 ± 50 | 55 ± 6 | 58% | 53 ± 46 | 60 ± 7 | 71% | ns |
| Saturated Fat (g/d) | 25 ± 12 | 24 ± 3 | 66% | 27 ± 22 | 26 ± 3 | 71% | ns |
| Sodium (mg/d) | 3336 ± 1444 ^a^ | 2300 ^b^ | 31% | 3004 ± 1484 ^a^ | 2300 ^b^ | 39% | *p* < 0.001 |

Mean ± Standard Deviation; ns= not significant; different letters indicate statistically significant differences (*p* < 0.001)

**Supplementary Table 2**

Food group intake, recommended values, and the percentage of the students meeting the recommendations.

|  | **Survey 1 (n = 62)** | | | **Survey 2 (n = 62)** | | |  |
| --- | --- | --- | --- | --- | --- | --- | --- |
| **Nutrient** | **Intake** | **Rec*** | **Percentage of Students Meeting Recommendation** | **Intake** | **Rec*** | **Percentage of Students Meeting Recommendation** | ***p-*value** |
| Grains (ounces) | 7 ± 4 | 7 ± 1 | 52% | 7 ± 4 | 8 ± 2 | 48% | ns |
| Fruits (cups) | 1 ± 1 ^a^ | 2 ± 0 ^b^ | 8% | 1 ± 1 ^a^ | 2 ± 0 ^b^ | 15% | *p* < 0.001 |
| Dairy (cups) | 1 ± 1 ^a^ | 3 ± 0 ^b^ | 8% | 1 ± 1 ^a^ | 3 ± 0 ^b^ | 6% | *p* < 0.001 |
| Vegetables (cups) | 2 ± 1 ^a^ | 3 ± 0 ^b^ | 18% | 1 ± 1 ^c^ | 3 ± 0 ^b^ | 5% | *p* < 0.001 |
| Total Fiber (g) | 15 ± 9 ^a^ | 27 ± 4 ^b^ | 21% | 13 ± 8 ^a^ | 30 ± 6 ^b^ | 11% | *p* < 0.001 |
| Proteins (g) | 99 ± 108 | 47 ± 3 | 94% | 79 ± 48 | 49 ± 5 | 74% | ns |

*Recommendation; Mean ± Standard Deviation; ns= not significant; different letters indicate statistically significant differences (*p* < 0.001)

**Supplementary Table 3**

Vitamin intake, recommended values, and percentage of the students meeting the recommendation.

|  | **Survey 1 (n = 62)** | | | **Survey 2 (n = 62)** | | |  |
| --- | --- | --- | --- | --- | --- | --- | --- |
| **Vitamin** | **Intake** | **Rec*** | **Percentage of Students Meeting Recommendation** | **Intake** | **Rec*** | **Percentage of Students Meeting Recommendation** | ***p-value*** |
| Folate (mcg) | 506 ± 295 | 400 ± 0 | 68% | 502 ± 289 | 400 ± 0 | 69% | ns |
| Niacin (mg) | 27 ± 16 ^a^ | 14 ± 1 ^b^ | 92% | 27 ± 16 ^a^ | 15 ± 1 ^b^ | 82% | *p* < 0.001 |
| Riboflavin (mg) | 2 ± 1 ^a^ | 1 ± 0 ^b^ | 92% | 2 ± 1 ^a^ | 1 ± 0 ^b^ | 81% | *p* < 0.001 |
| Thiamine (mg) | 2 ± 1 ^a^ | 1 ± 0 ^b^ | 85% | 2 ± 1 ^a^ | 1 ± 0 ^b^ | 77% | *p* < 0.001 |
| Vitamin A (mcg) | 475 ± 314 ^a^ | 723 ± 64 ^b^ | 19% | 396 ± 339 ^a^ | 774 ± 97 ^b^ | 18% | *p* < 0.001 |
| Vitamin B6 (mg) | 2 ± 2 ^a^ | 1 ± 0 ^b^ | 76% | 2 ± 2 ^a^ | 1 ± 0 ^b^ | 63% | *p* < 0.001 |
| Vitamin B12 (mcg) | 5 ± 4 ^a^ | 2 ± 0 ^b^ | 71% | 4 ± 3 ^a^ | 2 ± 0 ^b^ | 61% | *p* < 0.001 |
| Vitamin C (mg) | 59 ± 59 | 73 ± 9 | 37% | 49 ± 62 ^a^ | 80 ± 7 ^b^ | 21% | *p* < 0.001 |
| Vitamin D (IU) | 200 ± 198 ^a^ | 600 ± 0 ^b^ | 5% | 133 ± 147 ^a^ | 600 ± 0 ^b^ | 2% | *p* < 0.001 |
| Vitamin E (mg) | 8 ± 4 ^a^ | 15 ± 0 ^b^ | 13% | 7 ± 6 ^a^ | 15 ± 0 ^b^ | 15% | *p* < 0.001 |
| Vitamin K (mcg) | 114 ± 195 | 88 ± 10 | 52% | 98 ± 123 | 99 ± 15 | 32% | ns |

*Recommendation; Mean ± Standard Deviation; ns= not significant; different letters indicate statistically significant differences (*p* < 0.001)

**Supplementary Table 4**

Mineral intake, recommended values, and percentage of the recommendation met.

|  | **Survey 1 (n = 62)** | | | **Survey 2 (n = 62)** | | |  |
| --- | --- | --- | --- | --- | --- | --- | --- |
| **Mineral** | **Intake** | **Rec*** | **Percentage of Students Meeting Recommendation** | **Intake** | **Rec*** | **Percentage of Students Meeting Recommendation** | ***p-value*** |
| Calcium (mg) | 822 ± 555 ^a^ | 1044 ± 216 ^b^ | 34% | 692 ± 513 ^a^ | 1015 ± 65 ^b^ | 23% | *p* < 0.001 |
| Copper (mg) | 1 ± 0 | 1 ± 0 | 76% | 1 ± 1 | 1 ± 0 | 55% | ns |
| Iron (mg) | 14 ± 7 | 16 ± 3 | 42% | 13 ± 7 | 14 ± 5 | 50% | ns |
| Magnesium (mg) | 264 ± 136 ^a^ | 332 ± 32 ^b^ | 31% | 227 ± 124 ^a^ | 345 ± 44 ^b^ | 21% | *p* < 0.001 |
| Phosphorus (mg) | 1272 ± 618 ^a^ | 842 ± 243 ^b^ | 81% | 1114 ± 588 ^a^ | 727 ± 119 ^b^ | 79% | *p* < 0.001 |
| Potassium (mg) | 2266 ± 1057 | 2432 ± 371 | 47% | 2038 ± 977 ^a^ | 2850 ± 426 ^b^ | 26% | *p* < 0.001 |
| Selenium (mcg) | 133 ± 65 ^a^ | 55 ± 0 ^b^ | 97% | 123 ± 74 ^a^ | 55 ± 0 ^b^ | 84% | *p* < 0.001 |
| Zinc (mg) | 12 ± 7 | 9 ± 1 | 73% | 11 ± 8 | 9 ± 1 | 52% | ns |

*Recommendation; Mean ± Standard Deviation; ns= not significant; different letters indicate statistically significant differences (*p* < 0.001)

# Supplementary Figures

**Supplementary Figure 1** A: Consumption of the three main daily meals (breakfast, lunch, and dinner) among students surveyed at Finis Terrae University, Chile. B: Percentage of respondents reporting energy intake at recommended levels (green), under (red), or over recommended levels (brown). NA (Not Applicable) represents respondents who did not report their intake. Survey 1 (n=62); Survey 2 (n=62).

**Supplementary Figure 2**: Percentage of respondents reporting macronutrient intake at recommended levels (green), under (red), or over recommended levels (brown). NA (Not Applicable) represents respondents who did not report their intake. Survey 1 (n=62); Survey 2 (n=62).
